# Draft Genome Sequences for 6 Isolates of Endospore-Forming Class *Bacilli* Species Isolated from Soil from a Suburban, Wooded, Developed Space

**DOI:** 10.1128/mra.00874-22

**Published:** 2022-10-13

**Authors:** Anna L. McLoon, Thomas T. Awad, Molly F. Bogardus, Meredith G. Buono, Kaitlyn A. Devine, Rebecca M. Draper, Brianna Femenella, Hannah M. Gallagher, Laura A. Morelock, Mishal Razi, Jacqueline R. Rennick, Abigail K. Sheridan, Righlee J. Thibault, Katie L. Touchette, Grace E. Zuchowski

**Affiliations:** a Department of Biology, Siena College, Loudonville, New York, USA; Wellesley College

## Abstract

Here, we report draft genome sequences of Bacillus pseudomycoides SC107, *Rossellomorea* sp. SC111, Peribacillus frigoritolerans SC112, Priestia megaterium SC114, *Paenibacillus* sp. SC116, and Lysinibacillus fusiformis SC117, isolated from a campus wooded area in Loudonville, NY. Genomes were sequenced using the Illumina NextSeq system. The genomes range between 4,381,526 and 6,065,181 bp with GC contents between 35.33% and 43.46%.

## ANNOUNCEMENT

Soil bacteria and endospore-forming bacteria, in particular, play critical environmental roles in biomass degradation and plant health and many have also been sources for commercially important secondary metabolites, including antibiotics (1, 2). Sequencing multiple isolates of known species also can identify new genes and gene clusters not found in other members of the same species, increasing our understanding of microbial diversity. We collected a soil sample on 5 January 2022 from a heavily impacted wooded area between a parking lot and a marsh overgrown with *Phragmites* reeds on the Siena College campus (42°43′11.0″N, 73°44′59.6″W; 42.719711, −73.749876) near a willow tree (*Salix* sp.). The soil in the area is Stafford loamy fine sand and was frozen at the time of collection. Subsamples of the soil were suspended in water and boiled for 10 min to isolate endospores and then cultured on 5% sheep blood tryptic soy agar (TSA) plates overnight at 37°C. Six isolates were recultured and assessed via Gram staining and other tests to ensure purity. All isolates grew well on TSA at temperatures between 22 and 37°C. All 6 isolates were identified in this work as members of the *Bacilli* class (3), with several in newly reclassified genera (Table 1) (4, 5).

**TABLE 1:** Data summary

Strain	Species	Total no. of reads	No. of contigs	Total length (bp)	*N*_50_ (bp)	GC content (%)	No. of predicted genes (Prokka via KBase)
SC107	Bacillus pseudomycoides	6,363,180	97	5,714,291	270,478	35.33	5,885
SC111	*Rossellomorea sp.*	7,539,986	36	4,381,526	242,411	43,42	4,503
SC112	*Peribacillus frigoritolerans*	8,203,330	54	5,332,774	241,338	40.51	5,193
SC114	*Priestia megaterium*	7,160,128	38	6,065,181	4,198,608	37.45	6,305
SC116	*Paenibacillus sp.*	7,022,018	22	5,444,016	2,729,508	43.46	4,852
SC117	Lysinibacillus fusiformis	8,267,586	30	4,809,961	474,952	37.09	4,670

For genomic DNA isolation, cells were grown in tryptic soy broth at 37°C for 3 to 4 h, until the culture was visibly dense. Cells were resuspended in 50 mM EDTA, and peptidoglycan was digested for 1 h at 37°C with the addition of 30 μL of 20 mg/mL lysozyme, and then genomic DNA was purified using a Promega Wizard genomic DNA purification kit. Libraries were prepared using the Illumina DNA prep kit and IDT 10-bp unique dual indexes (UDIs) and were sequenced as 151-bp paired-end reads using an Illumina NextSeq2000 instrument by the Microbial Genome Sequencing Center, Pittsburgh, PA (Table 1), who also carried out demultiplexing, quality control, and adapter trimming using bcl-convert v3.9.3 (Illumina). Subsequent analysis steps were carried out by the authors within the KBase environment (6, 7). Analyses were carried out in parallel for each isolate in its own narrative. Sequence quality was validated with FastQC v0.11.9 (8), and then reads were trimmed with Trimmomatic v0.36 (9). Trimmed reads were assembled into genome assemblies using SPAdes v3.15.3 (10, 11) and were annotated using RAST v1.073 (12–14) and/or Prokka v.1.14.5 (15), the species identity was assigned using GTDB-Tk v1.7.0 (16–18) and Type Strain Genome Server (TYGS) (19, 20) (Table 1), and metabolic predictions were made using DRAM v0.1.0 (21). All 6 genomes were also annotated automatically with PGAP v6.2 when deposited to GenBank (22–24). Default parameters were used throughout. Genome assemblies range in size from 4.38 to 6.07 Mbp and are AT rich (Table 1). These genomes provide a basis for future comparative genomics studies and investigation of secondary metabolite production by soil endospore-forming bacteria.

### Data availability.

The genome sequence and raw sequencing reads were deposited under BioProject accession number PRJNA862062; Sequence Read Archive (SRA) accession numbers SAMN29936620, SAMN29936621, SAMN29936622, SAMN29936623, SAMN29936624, and SAMN29936625; and GenBank accession numbers JANIOB000000000, JANIOC000000000, JANIOD000000000, JANIOE000000000, JANIOF000000000, and JANIOG000000000. Analyses are available through KBase narratives 110380, 110406, 110408, 110407, 110414, and 109725, and the overview narrative is 122484.
